# Genomic epidemiology of animal-derived tigecycline-resistant *Escherichia coli* across China reveals recent endemic plasmid-encoded *tet*(X4) gene

**DOI:** 10.1038/s42003-020-01148-0

**Published:** 2020-07-31

**Authors:** Chengtao Sun, Mingquan Cui, Shan Zhang, Dejun Liu, Bo Fu, Zekun Li, Rina Bai, Yaxin Wang, Hejia Wang, Li Song, Chunping Zhang, Qi Zhao, Jianzhong Shen, Shixin Xu, Congming Wu, Yang Wang

**Affiliations:** 1 Beijing Key Laboratory of Detection Technology for Animal Food Safety, College of Veterinary Medicine, China Agricultural University, Beijing, China; 2 China Institute of Veterinary Drug Control, Beijing, China

**Keywords:** Antimicrobial resistance, Bacterial genes

## Abstract

Public health interventions to control the recent emergence of plasmid-mediated tigecycline resistance genes rely on a comprehensive understanding of its epidemiology and distribution over a wide range of geographical scales. Here we analysed an *Escherichia coli* collection isolated from pigs and chickens in China in 2018, and ascertained that the *tet*(X4) gene was not present at high prevalence across China, but was highly endemic in northwestern China. Genomic analysis of *tet*(X4)-positive *E. coli* demonstrated a recent and regional dissemination of *tet*(X4) among various clonal backgrounds and plasmids in northwestern China, whereas a parallel epidemic coincided with the independent acquisition of *tet*(X4) in *E. coli* from the remaining provinces. The high genetic similarity of *tet*(X4)-positive *E. coli* and human commensal *E. coli* suggests the possibility of its spreading into humans. Our study provides a systematic analysis of the current epidemiology of *tet*(X4) and identifies priorities for optimising timely intervention strategies.

## Abstract (web summary)

Sun et al. analyse *E. coli* strains from pigs and chickens in Chinese farms and slaughterhouses, and reveal that while the *tet*(X4) gene is not ubiquitous across China, it is highly endemic in northwestern China. Genomic analysis of these strains shows recent and independent acquisitions of plasmids in across regions.

## Introduction

The incessant emergence of novel and transmissible antimicrobial resistance (AMR) mechanisms has frustrated clinicians because limited antimicrobial agents are left for the treatment of infectious diseases^1^. Classified by the World Health Organization as a critically important antimicrobial agent, tigecycline provides a key line of defense against multi-drug-resistant bacteria, particularly in cases of life-threatening infections^2^. Up until 2019, reported resistance to tigecycline was commonly mediated by mutational and regulatory changes with limited lateral transferability^3,4^. However, plasmid-encoded *tet*(X) genes that confer high-level tigecycline resistance were first described in isolates from animals and humans in China in around 2018^5–7^. Subsequently, several more *tet*(X) variants—*tet*(X3), *tet*(X4), and *tet*(X5)—were identified on transferable plasmids in *Enterobacteriaceae* and *Acinetobacter* isolates from animals and humans in China^5,6^. In addition to their ability to degrade all tetracyclines, the novel plasmid-mediated *tet*(X) variants show a much more efficient horizontal transferability, making the host bacteria and recipient pathogens a new and severe threat to global public health^5–7^.

Initial epidemiological studies in China indicated that the current presence of *tet*(X3) and *tet*(X4) is still rare in the human sector, but more common in food animals, meat products, and the surrounding niches^5–8^, suggesting that the food animals may be large reservoir of these novel mobile tigecycline resistance genes. A further retrospective study reported that the emergence of *tet*(X4) in food animals is a recent event^9^, but is now spreading geographically^5–8,10^, indicating an emerging risk of food animals as reservoirs in spreading these genes. Public health interventions to control the spread of AMR genes rely on a comprehensive understanding of its current epidemiology and distribution over a wide range of geographical scales. However, tigecycline is not authorized in food animals, its resistance in animals is not routinely monitored and largely unknown. Given the clinical importance of tigecycline and the emerging risk of food animals in spreading its resistance, a further comprehensive surveillance is urgently recommended to underline the current situation of these newly identified *tet*(X) variants^5–10^.

Here, we analysed an *E. coli* collection isolated from pigs and chickens in China 2018. We ascertained that the gene *tet*(X4) was not present in high prevalence on a China-wide scale, but was highly endemic in northwestern China. We illustrated a complex combination of multiple genetic vehicles (mobile genetic elements, plasmids and bacterial lineages) in the spreading of the *tet*(X4) gene, and showed a possibility of *tet*(X4)-positive *E. coli* in spreading into the humans.

## Results

### *tet*(X4) was endemic in northwestern China

**Fig. 1:**
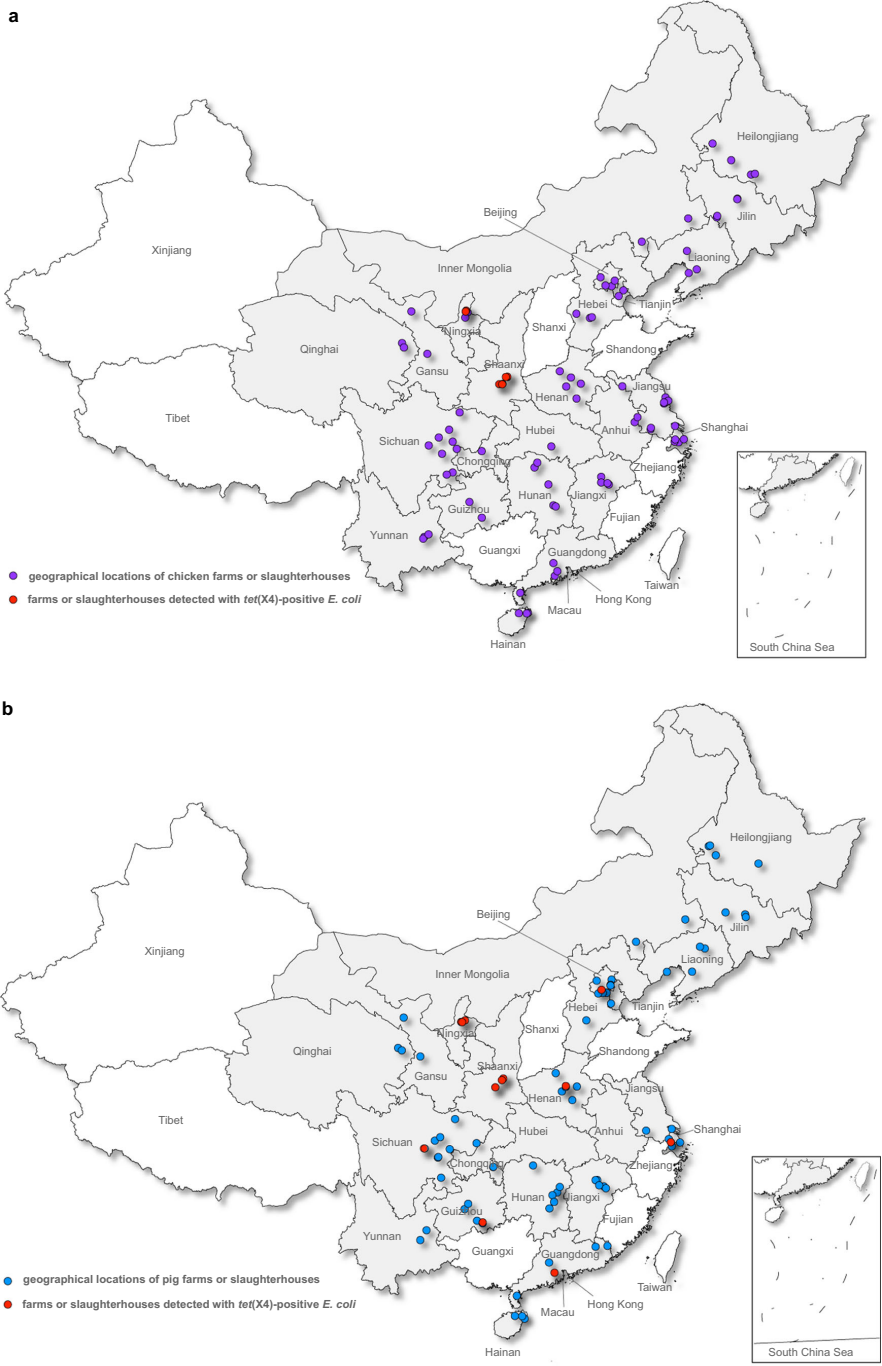
Geographical distribution of farms and slaughterhouses presenting *E. coli* isolates in 2018 in China. **a***E. coli* isolates from chickens (*n* = 1245). **b***E. coli* isolates from pigs (*n* = 1230). Provinces and municipalities included in the study are shaded in grey, dots on the maps indicate locations of farms or slaughterhouses in included in the study, red dots indicate the farms or slaughterhouses tested positive for *tet*(X4).

From the 2475 *E. coli* isolates included in the study, 125 tigecycline-non-susceptible *E. coli* were recovered. The *tet*(X4) gene was detected in 95 isolates originating from eight provinces, including 60 pig isolates (60/1,230, 4.9%, 95% confidence interval (CI): 3.7–6.2%) and 35 chicken isolates (35/1,245, 2.8%, 95% CI: 2.0–3.9%) (Table 1 and Fig. 1). Shaanxi and Ningxia, two neighbouring provinces in northwestern China, had a relatively high *tet*(X4) prevalence in both pigs (30/60, 50.0%, Shaanxi; 20/45, 44.4%, Ningxia) and chickens (20/45, 44.4%, Shaanxi; 15/45, 33.3%, Ningxia). In contrast, the *tet*(X4) gene appeared sporadically among pig isolates from Sichuan (4/150, 2.7%), Henan (2/75, 2.7%), Guizhou (1/60, 1.7%), Beijing (1/135, 0.74%), Shanghai (1/75, 1.3%), and Guangdong (1/75, 1.3%) and was negative in the remaining 14 provinces. The chicken isolates that were positive for *tet*(X4) were only detected from Shaanxi and Ningxia (Table 1 and Fig. 1). All amplified fragments had 100% sequence homology to the original *tet*(X4) gene^5^. The *tet*(X3) and *tet*(X5) genes were absent in the current strain collection. The underlying tigecycline resistance mechanisms of the 30 tigecycline-non-susceptible *E. coli* that were all negative for novel *tet*(X) variants were not explored in the current study and will be discussed elsewhere.

**Table 1:** Distribution of *E. coli* and *tet*(X4)-positive *E. coli* from pigs and chickens in China in 2018.

Province	No. of *E. coli* isolates		No. of positive *E. coli*		*%* of positive *E. coli* (95% CI)	
	Pig	Chicken	Pig	Chicken	Pig	Chicken
Beijing	135 (9)^a^	60 (4)	1 (1)	0	0.74%	0.00%
Chongqing	30 (2)	15 (1)	0	0	0.00%	0.00%
Gansu	30 (2)	30 (2)	0	0	0.00%	0.00%
Guangdong	75 (5)	60 (4)	1 (1)	0	1.33%	0.00%
Guizhou	60 (4)	30 (2)	1 (1)	0	1.67%	0.00%
Hainan	45 (3)	45 (3)	0	0	0.00%	0.00%
Hebei	15 (1)	45 (3)	0	0	0.00%	0.00%
Heilongjiang	60 (4)	60 (4)	0	0	0.00%	0.00%
Henan	75 (5)	75 (5)	2 (1)	0	2.67%	0.00%
Hunan	60 (4)	90 (6)	0	0	0.00%	0.00%
Inner Mongolia	30 (2)	15 (2)	0	0	0.00%	0.00%
Jiangsu	15 (1)	150 (10)	0	0	0.00%	0.00%
Jiangxi	75 (5)	60 (4)	0	0	0.00%	0.00%
Jilin	45 (3)	60 (4)	0	0	0.00%	0.00%
Liaoning	75 (5)	45 (3)	0	0	0.00%	0.00%
Ningxia	45 (3)	45 (3)	20 (3)	15 (2)	44.44%	33.30%
Qinghai	30 (2)	30 (2)	0	0	0.00%	0.00%
Shaanxi	60 (4)	45 (3)	30 (4)	20 (3)	50.00%	44.40%
Shanghai	75 (5)	75 (5)	1 (1)	0	1.33%	0.00%
Sichuan	150 (10)	135 (9)	4 (1)	0	2.67%	0.00%
Tianjin	15 (1)	30 (2)	0	0	0.00%	0.00%
Yunnan	30 (2)	45 (3)	0	0	0.00%	0.00%
Total	1230 (82)	1245 (84)	60 (13)	35 (5)	4.88% (3.74–6.23%)	2.81% (1.97–3.89%)

^a^Numbers in parentheses are numbers of farms and slaughterhouses.

### The antimicrobial resistant phenotype and genotype

Susceptibility testing confirmed that 95 *tet*(X4)-positive *E. coli* showed resistance to tigecycline (minimum inhibitory concentrations (MICs) 4–32 mg L^−1^), doxycycline (32–128 mg L^−1^), and florfenicol (32–128 mg L^−1^) but exhibited sensitivity to meropenem. A few of the isolates showed resistance to colistin (*n* = 3, 3.2%), cefepime (*n* = 1, 1.1%), ceftriaxone (*n* = 4, 4.2%), aztreonam (*n* = 5, 5.3%), and gentamicin (*n* = 7, 7.4%), while the majority were resistant to ampicillin (*n* = 92, 96.8%) and amoxicillin-clavulanate (*n* = 94, 98.8%) (Supplementary Data 1).

Apart from *tet*(X4), a median of 13 AMR genes (range, 2–20) was detected in the genome of each isolate. Genes coding for resistance to phenicols (*floR*, 91/95), tetracyclines (*tet*(A), 87/95), sulfonamides (*sul2*, 26/39; *sul3*, 71/95), aminoglycosides (*strA*, 81/95; *strB*, 81/95; *aadA2*, 70/95), and trimethoprims (*drfA12*, 69/95) were highly present in the *E. coli* harbouring *tet*(X4). The colistin resistance gene *mcr-1* was detected in two of the 95 *E. coli* isolates (Supplementary Fig. 1 and Supplementary Data 2). All *tet*(X4)-positive *E. coli* were classified into five phylogroups (A, *n* = 79; B1, *n* = 12; B2, *n* = 1; C, *n* = 1, and F, *n* = 2). One pig strain SC-P337 (Sichuan, ST4541, O146:H28) was placed into group B2, which is commonly known as being highly pathogenic, and carried a greater number of virulence-factor-associated genes (*n* = 62) than isolates from group A (Supplementary Fig. 1 and Supplementary Data 2).

### Genomic diversity of *tet*(X4)-positive *E. coli*

**Fig. 2:**
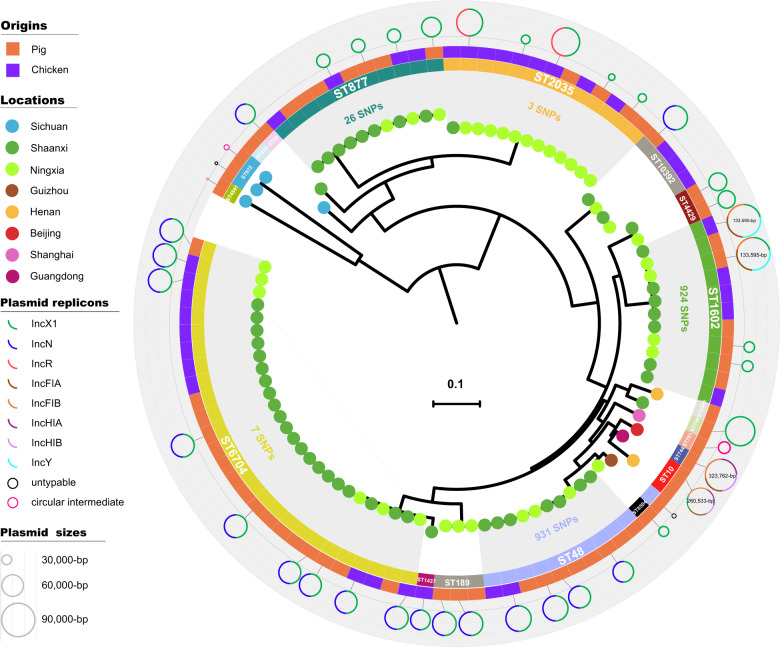
Phylogenetic tree of the 95 *tet*(X4)-positive *E. coli* analysed in this study. Neighbour-Joining tree constructed based on 162,383 core-genomic SNPs. Each isolate is labelled on the node with a coloured dot in representing its location, the STs are labelled and shown in the inner ring (the five major lineages are indicated with grey), the origins of the isolates are indicated by coloured squares in the outer ring, and the 42 circles differing in coloured components representing the completely sequenced plasmids and circular intermediates in connecting each isolate. Squares with no connected plasmid circles are isolates not selected for ONT long-read sequencing.

In silico multilocus sequence typing (MLST) clustered the 95 *tet*(X4)-positive *E. coli* into 19 distinct sequence types (STs), with 60 pig isolates clustered in 18 STs and 35 chicken isolates in seven STs (Supplementary Fig. 2). Although diversified in STs, the majority (76.8%, 73/95) of the isolates were concentrated into five major STs, 6704 (27/95), 2035 (13/95), 48 (11/95), 1602 (11/95), and 877(11/95), which mostly (73/74) originated from Shaanxi and Ningxia (Fig. 2 and Supplementary Fig. 2). Isolates within each major ST were characterized by low levels of core genome diversity that was reflected by a relatively limited number of single nucleotide polymorphisms (SNPs) (differing in 3–931 SNPs) (Fig. 2). However, isolates (*n* = 10) from the remaining six provinces and municipalities showed relatively greater genetic diversity among each other, composing 8 of the 19 STs (Fig. 2).

### Versatile plasmids harbouring *tet*(X4)

Based on the phylogenetic analysis, around one-third of the isolates within each of the major lineages were selected (*n* = 25), together with most of the remaining isolates (*n* = 17), for Oxford Nanopore Technologies (ONT) long-read sequencing (Supplementary Data 2). Hybrid de novo assembly produced 39 completely sequenced plasmids possessing *tet*(X4) and three circular intermediates (CIs) harbouring *tet*(X4) but without replicons. Incompatibility typing of the 39 plasmids (ranging in sizes from 8581 to 322,239 bp) showed a striking variety of plasmid types. In addition to a handful of IncX1 plasmids (*n* = 13; 21,050–82,762 bp) and two untypable plasmids (8581 and 12,805 bp), a majority of the plasmids (25/39) were detected with multi-replicons, including IncX1-IncN (*n* = 18; 55,530–71,790 bp), IncX1-IncR (*n* = 2; 76,546 bp and 77,171 bp), IncX1-IncFIA/B-IncY (*n* = 2; 133,595 bp and 133,595 bp), IncX1-IncFIA/B-IncHI1A/B (*n* = 1; 260,533 bp), and IncFIA/B-IncHI1A/B (*n* = 1; 323,762 bp) (Fig. 2 and Supplementary Data 2). Plasmids of the same Inc type could be identified not only from isolates of the same lineage, but also from isolates of the different lineages, indicating the spread of the *tet*(X4) gene is combining vertical and horizontal transferability (Fig. 2). Outside northwestern China, plasmids exhibited limited comparability but endemism to those harboured in the major lineages, such as the two large multi-replicon plasmids in the isolates from Beijing (ST744, IncFIA/B-IncHI1A/B) and Guangdong (ST10, IncX-IncFIA/B-IncHI1A/B), as well as the small untypable plasmids from Sichuan (ST792) and Guizhou (ST48); however, the endemic IncX1 and IncX1-IncN plasmids from northwestern China were not observed elsewhere (Fig. 2).

### MGE arrangements in spreading *tet*(X4)

**Fig. 3:**
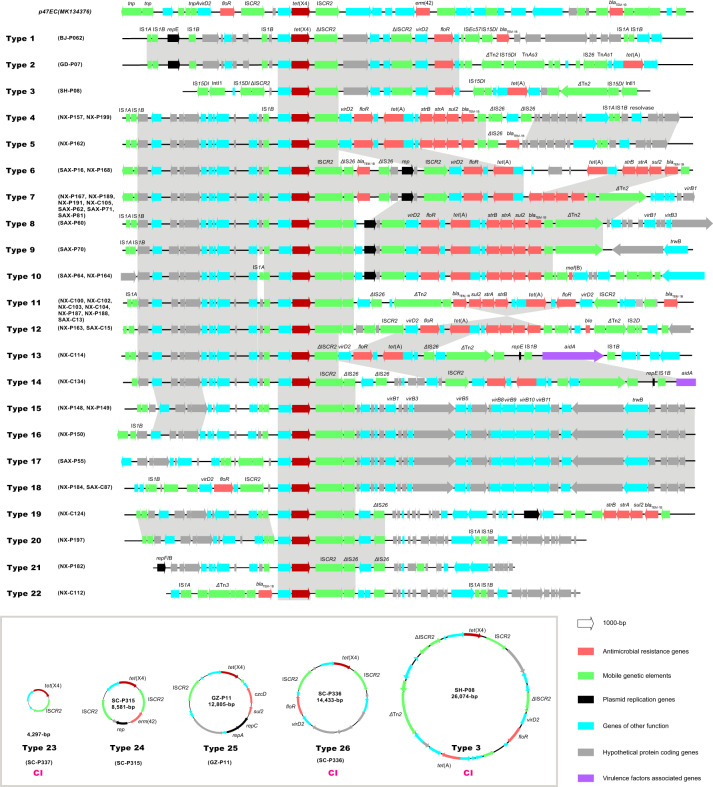
Genetic environment of *tet*(X4) in the 39 completely sequenced plasmids and 3 circular intermediates. The arrows indicate the direction of transcription of the genes, and the genes are differentiated by colours. Regions of >99% homology are marked by grey shading.

**Fig. 4:**
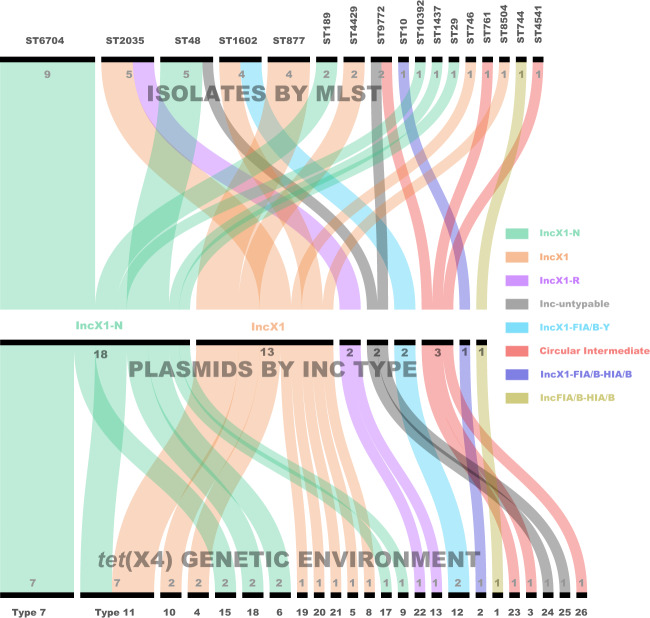
Sankey diagram combining the isolates, plasmids, and genetic environments bearing *tet*(X4). The lines are drawn connecting STs, plasmid Inc types, and *tet*(X4) genetic environments based on corresponding information from the 39 completely sequenced plasmids and 3 circular intermediates as well as the host strains. The diameter of the line is proportional to the number of isolates, which is also labelled at the consolidation points. Lines are coloured based on plasmid Inc types.

To further analyse the transfer of *tet*(X4) via mobile genetic elements (MGE), the sequenced plasmids were probed for *tet*(X4) genetic contexts and were subsequently correlated to plasmid types and the host strains. The genetic environments of *tet*(X4) were clustered into 26 types, differing mostly in the variable context downstream of *tet*(X4) but showing a relatively conserved architecture upstream of the gene (Fig. 3). Compared with the flanking stance of the two IS*CR2* copies in the original *tet*(X4)-harbouring plasmid p47EC (IS*CR2*-ORF2-*abh*-*tet*(X4)-IS*CR2*)^5^, the upstream copy of IS*CR2* adjacent to *abh*-*tet*(X4) was absent in most of the present types (20/26) but generally replaced by a truncated IS*1B* element (IS*1B*-*abh*-*tet*(X4)-IS*CR2*). In contrast, the downstream copy of IS*CR2* remained tightly associated with *tet*(X4), forming a unified central region *abh*-*tet*(X4)-IS*CR2* (Fig. 3). A further downstream search generally found a second copy of IS*CR2* (12/26, type 1–3, 6–12, 14, and 19), which was joined by the type IV secretion system component *virD2* relaxase and led to a set of other resistance determinants, mainly a gene cluster of *floR*, *tet*(A), *strB*, *strA*, *sul2*, *bla*_TEM-1B_, and occasionally *mef*(B) and *ble*. Whenever the second copy of IS*CR2* was absent*, virD2* and the adherent resistance genes were linked directly to the first copy of IS*CR2* downstream of *tet*(X4) (type 4, 5, 13, 24, and 26) (Fig. 3). By connecting the derived genetic environment types back to plasmids (seven Inc types) and the host strains (16 STs), we illustrated a complex combination of multiple genetic vehicles in the spreading of the *tet*(X4) gene (Fig. 4).

### The plasmid resistome in connecting *tet*(X4)

**Fig. 5:**
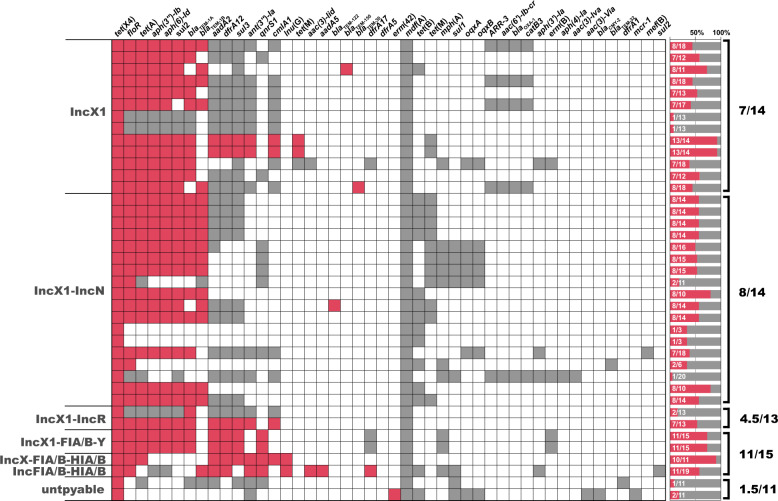
Heatmap showing the incidence of AMR genes within the 42 completely sequenced *tet*(X4)-positive *E. coli*. Each row in the heat map represents one isolate. Isolates are grouped by incompatibility types of the *tet*(X4)-harbouring plasmids. Coloured cells in each row indicate the presence of a particular resistance gene (as labelled at the top). Red cells indicate AMR genes located on the *tet*(X4)-harbouring plasmids; grey cells indicate AMR genes in the genome but not on the *tet*(X4)-harbouring plasmid. Bars on the left indicate the percentage of AMR genes located on *tet*(X4)-harbouring plasmids from that of the whole genome. The fractions indicate the median number of AMR genes on *tet*(X4)-harbouring plasmid/AMR genes within the whole genome.

Given the fact of co-selection in driving plasmids’ persistence, we further examined the resistome of *tet*(X4)-harbouring plasmids. A median of eight different AMR genes (range, 1–13) was detected within the 39 completely sequenced plasmids, while the median number of AMR genes was 14 (range, 3–20) when scrutinizing the 39 whole genomes. Indeed, we found that a majority of *tet*(X4)-positive plasmids (23/39) possessed over half of the whole genomes’ resistance genes (Fig. 5), indicating the central role of *tet*(X4)-positive plasmids in facilitating the host bacteria’s resistance to antimicrobials.

The phenicol resistance gene *floR* represented the highest occurrence (31/39), followed by genes conferring resistance to tetracyclines (*tet*(A), 28/39), aminoglycosides (*aph(3”)-Ib*, 27/41 and *aph(6)-Id*, 27/39), sulfonamides (*sul2*, 26/39), and β-lactams (*bla*_TEM-1A,_ 25/39 and *bla*_TEM-1B_, 18/39). Generally, high numbers of resistance genes were observed in the syncretic plasmids with three or more replicons (median, 11) (Fig. 5 and Supplementary Data 2).

### High genetic propensity of *tet*(X4) in spreading into humans

**Fig. 6:**
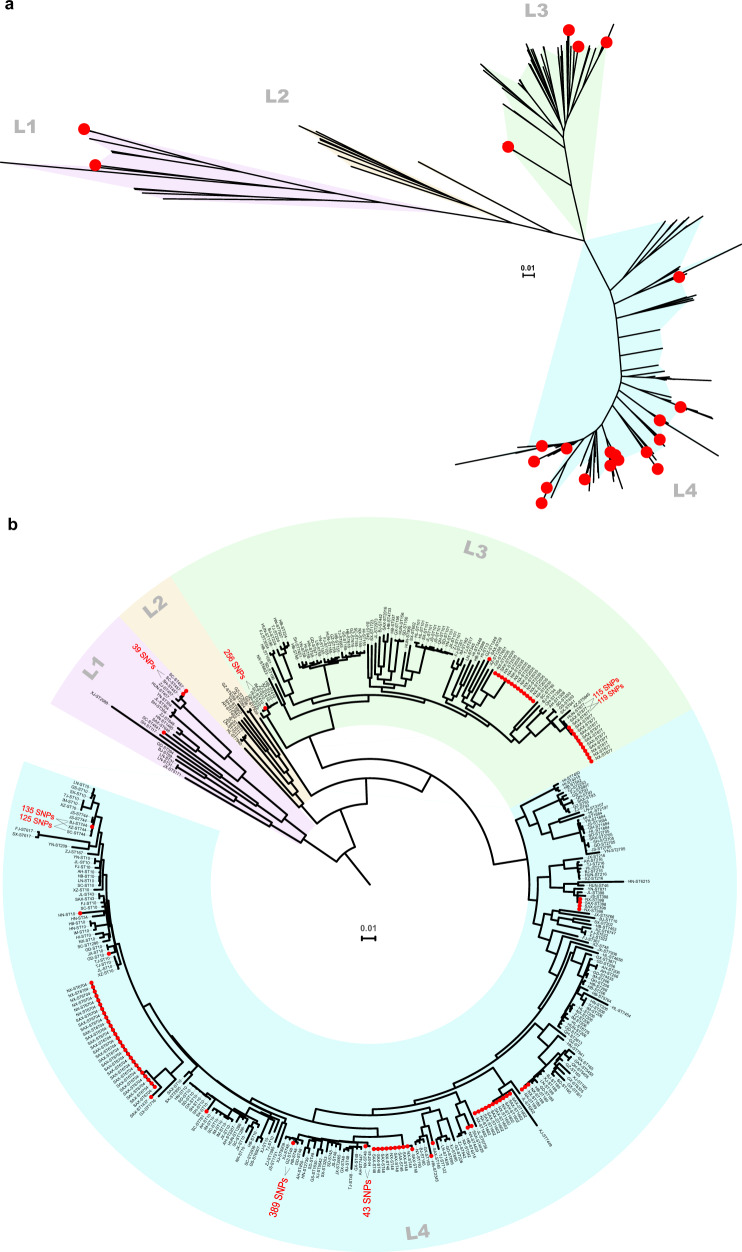
High genetic propensity of *tet*(X4)-harbouring *E. coli* in spreading into humans. Phylogenetic trees of the 95 *tet*(X4)-positive *E. coli* isolates together with 287 publicly available draft genomes of *tet*(X4)-negative *E. coli* (PRJNA400107) from healthy humans across China (Neighbour-Joining trees constructed based on 166,544 core-genome SNPs). Red dots represent the 95 isolates positive for *tet*(X4). **a** An unrooted tree showing the four Bayesian lineages. **b** A circular tree showing the corresponding information and SNP differences between isolates that are positive or negative for *tet*(X4). Abbreviations: AH, Anhui; BJ, Beijing; FJ, Fujian; GS, Gansu; GD, Guangdong; GX, Guangxi; GZ, Guizhou; HI, Hainan; HB, Hubei; HUN, Hunan; HN, Henan; HL, Heilongjiang; JL, Jilin; JS, Jiangsu; JX, Jiangxi; LN, Liaoning; IM, Inner Mongolia; NX, Ningxia; QH, Qinghai; SD, Shandong; SX, Shanxi; SAX, Shaanxi; SH, Shanghai; SC, Sichuan; TJ, Tianjin; XZ, Xizang; XJ, Xinjiang; Yunnan; ZJ, Zhejiang.

To determine the genetic propensity of *tet*(X4)-positive isolates to spread into the human sector, we analysed the genetic relatedness of our 95 *tet*(X4)-positive *E. coli* to 287 publicly available draft genomes of human commensal *E. coli* (PRJNA400107) that were negative for *tet*(X4). The negative isolates were non-duplicates and originated from healthy individuals across China in 2016 as part of a previous study characterising *mcr-1* carriage in humans^11^. The 95 *tet*(X4)-harbouring *E. coli* were phylogenetically clustered into three of the four Bayesian lineages generated from the human isolates (L1, *n* = 3; L3, *n* = 26, and L4, *n* = 66) (Fig. 6a). A large proportion of our animal-derived *tet*(X4)-harbouring *E. coli* displayed high genetic similarity (25/95, as small as 39 SNPs) to the *E. coli* of human origin (Fig. 6b) from various provinces (Supplementary Fig. 3). Of particular concern is that the pig isolate from Shaanxi differed by only 119 SNPs from the human *E. coli* isolated in this province.

## Discussion

Data remain scarce on the newly identified plasmid-mediated tigecycline resistance genes. Combining a national *E. coli* collection and advances in bioinformatics, the present study reveals a comprehensive understanding of the *tet*(X4) gene in *E. coli* from food-producing animals across China. It is evident from our data that the *tet*(X4) gene was present at low prevalence at the national scale in 2018, but was highly endemic in northwestern China. The observed limited genetic diversity of the isolates from northwestern China and the promiscuous plasmids carrying *tet*(X4) indicate recent and regional dissemination and variably successful clonal backgrounds, thus further extending our previous commentary describing the recent emergence of the *tet*(X4) gene in China^9^. Although scattered *tet*(X4)-positive isolates were identified in the remaining six provinces, the distinct STs and plasmids observed suggest that a parallel epidemic has coincided with the independent acquisition of *tet*(X4)-harbouring plasmids. Given the extensive food animal husbandry and free trade within China, these *tet*(X4)-harbouring “sparks” are presumably spread over a wide range of geographical scales, as exemplified by the clonal and lateral expansions in northwest China. In this context, it is worrisome to speculate that a national pandemic of tigecycline-resistant *E. coli* in food animals is only a matter of time unless there is timely and effective eradication of these “sparks” before the “prairie fire”.

Moreover, our concerns are escalated by the high genetic propensity of *tet*(X4)-positive *E. coli* for spreading into humans. Although the phylogenetic analysis was based on human *E. coli* that positive for *mcr-1*, there were reports of *E. coli* coharbouring *tet*(X4) and *mcr-1*^6,9,12^. The high genetic similarity of *E. coli* between pigs and humans in the endemic region (Shaanxi province) indicates a possibility of spillover of the *tet*(X4) gene from animals to humans. In this circumstance, in vivo transfer of the *tet*(X4) gene into human pathogens would be highly likely to occur. This process might be co-selected and magnified by extensively used antibiotics in humans, such as β-lactams and aminoglycosides, as their resistant genes were observed in this study frequently accompanied by *tet*(X4).

The *tet*(X4)-harbouring plasmids resided in a wide variety of ST clones (Fig. 2 and Supplementary Fig. 2), but only three *E. coli* Bayesian lineages were implicated (Fig. 6a), thus suggesting a considerably narrow host spectrum of the plasmids carrying this gene. This is likely a similar scenario reminiscent of *mcr-1*, which resided primarily on IncX4 plasmids that flowed among four lineages (although diversified in 135 STs) of *E. coli* across China^11^, but it contrasts with the transmission of the *bla*_NDM_ gene among eight distinct *E. coli* lineages in a single Chinese poultry production chain^13^. However, the *tet*(X4) gene was identified in a large proportion of multireplicon plasmids, which are diversified in replicon gene combination. This is unusual, but not without precedent in that *mcr-1* has already been found in several hybrid versions of plasmids^11,14,15^, suggesting the involvement of plasmid fusion in the spreading of *tet*(X4). The presence of multiple replicons in *tet*(X4)-positive plasmids might prevent plasmid incompatibility^16^ and facilitate their interaction with a broad range of hosts^17^. Our plasmid resistome analysis confirmed that *tet*(X4)-positive plasmids are indeed versatile in spreading AMR genes. Further dissemination of these “super plasmids” into clinical settings would be a big threat. However, it remains to be seen whether the plasmids carrying *tet*(X4) are more prone to hybridisation, but the host ranges, the capacity to capture AMR genes, and the evolution of these new *tet*(X4) genetic platforms will need to be closely monitored.

Apart from a certain documented genetic environment of *tet*(X4), we have here expanded its habitation to a broader territory. IS*CR2* was found either flanked at both ends of *abh*-*tet*(X4) or existing solely downstream of *tet*(X4). Different from the transposon paradigm, a single copy of the IS*CR2* would transpose the adjacent sequences via rolling-circle transposition, which has been documented in the mobilization of several resistance genes^18^. In these circumstances, IS*CR2*-mediated transposition might play an essential role in the integration of *tet*(X4) into multiple plasmids, consequently enlarging the possibility of *tet*(X4) to propagate into the broad host range plasmids as well as other bacterial species. Taken together, the contribution of MGEs, in particular IS*CR2*, to the spread of *tet*(X4) gene should not be minimised.

Despite the findings, this work has several limitations that should be noted. First, we included approximately 15 *E. coli* randomly selected from each farm or slaughterhouse, which might not be proportional to the annual production (pig and chickens) of each province or municipality. Second, the direct screening of *tet*(X4) from *E. coli* identified without targeted tigecycline phenotype and enrichment steps might underestimate the true prevalence and diversity of *E. coli* carrying *tet*(X4), as previously seen in the detection of *mcr-1*^11^. Third, the majority of the *tet*(X4)-positive *E. coli* originated from Shaanxi and Ningxia, which might have biased the deduced results.

On a national scale, the current study shows the countenance of the newly emerged *tet*(X4)-mediated tigecycline resistance, which is undeniably a great public health threat. Based on these results, the intervention priorities should focus on (1) the rapid and thorough eradication of this gene in food animals in northwest China, (2) tight monitoring of this gene in humans, particularly in people who are epidemiologically related to northwestern China, and (3) continuous national surveillance and risk assessment of this gene in a broader One Health approach. Furthermore, our results may also serve as a benchmark for the current status of *tet*(X4)-positive *E. coli* at an early stage, to which future survey data can be compared, thus facilitating not only the understanding of the paradigmatic shift of resistance, but also critical intervention improvements.

## Methods

### Strain collections

This study is based on *E. coli* collections from China’s national AMR surveillance programme in zoonotic bacteria. The *E. coli* isolates were collected and submitted by provincial laboratories or institutes certified as part of the surveillance programme. The participation of farms and slaughterhouses in the surveillance system in each province was on a voluntary basis. Commensal *E. coli* isolates were identified without any targeted AMR phenotypes from healthy chickens (cloacal swabs) and pigs (faecal swabs). As of July 2019, the system had received a total of 3124 *E. coli* isolates in 2018 originating from 233 farms and slaughterhouses across 22 of the 34 provinces and municipalities in China. Because there was a large variation in the number of isolates from each farm and slaughterhouse, we randomly selected about 15 *E. coli* isolates from each farm or slaughterhouse in order to minimise the sample bias in the surveillance data, and this resulted in a total of 2,475 *E. coli* isolated from 166 farms and slaughterhouses distributed across the country (Fig. 1 and Table 1). Since no live vertebrates were used in this study, the included *E. coli* collection is exempt from the IACUC approval process.

### PCR-based screening of novel *tet*(X) variants

*E. coli* isolates were inoculated on MacConkey plates supplemented with tigecycline (2 mg/mL), and the resulting tigecycline-non-susceptible isolates were then subjected to *tet*(X) and variant screening using a two-step PCR analysis. A universal primer pair (*tet*(X) forward 5′-CCG TTG GAC TGA CTA TGG C-3′; *tet*(X) reverse 5′-TCA ACT TGC GTG TCG GTA A-3′) targeting a 475 bp conserved region of *tet*(X) and the four *tet*(X) variants was used, and the positives were further screened for *tet*(X3), *tet*(X4), and *tet*(X5) using primer pairs as previously described^5,7^. The genomic DNA of *tet*(X3)-carrying *Acinetobacter baumannii* 34AB, *tet*(X4)-carrying *E. coli* 47EC, and *tet*(X5)-carrying *A. baumannii* AB17H194 from previous studies were used as positive controls^5,7^.

### Antimicrobial susceptibility testing

MICs were determined for all *tet*(X4)-carrying *E. coli* using broth microdilution in Mueller-Hinton broth (Oxoid, Basingstoke, UK). The tested antibiotics included tigecycline, colistin, meropenem, cefepime, ceftriaxone, ampicillin, amoxicillin-clavulanate, aztreonam, ciprofloxacin, gentamicin, doxycycline, and florfenicol. Susceptibility was determined according to the European Committee on Antimicrobial Susceptibility Testing (EUCAST, version 10.0, for tigecycline, colistin, and florfenicol)^19^ and the Clinical and Laboratory Standards Institute document (CLSI, M100-S29, for the remaining antibiotics)^20^. Reference strain *E. coli* ATCC 25922 and *Staphylococcus aureus* ATCC 29213 served as the quality control strains.

### Whole-genome sequencing and assembly

All positive *E. coli* isolates were subjected to whole-genome sequencing. Briefly, a KAPA Hyper Prep Kit (Kapa Biosystems, Boston, MA, US) was used for library construction, and 300-bp paired-end reads with a minimum of 150-fold coverage for each isolate were obtained following sequencing using the Illumina HiSeq X Ten System. A draft assembly of the cleaned reads was generated using SPAdes version 3.9.0^21^. To determine the plasmid or chromosome location and the genetic environment of the *tet*(X4) gene, a number of isolates were selected based on phylogenetic analysis and background information regarding the source for further sequencing by ONT long-read sequencing. Briefly, libraries were prepared using the Rapid Barcoding Kit (SQK-RBK004) and subjected to ONT long-read sequencing in R9.4.1 flow cells in a MinION sequencer according to the standard protocol A 0.3.4. The resulting long reads were subjected to hybrid de novo assembly in combination with Illumina short reads^22^.

### Phylogenetic analysis

Core-genome SNP-based Neighbour-Joining phylogenetic trees were constructed for all sequenced isolates using Parsnp in the Harvest package^23^ with default parameter settings, and these were visualised using iTOL v5^24^ with the corresponding features of each isolate. To determine the genetic plasticity of the *tet*(X4) gene in its spread into the humans, we analysed the genetic relatedness of all *tet*(X4)-positive *E. coli* in the current study together with 287 publicly available draft genomes of *E. coli* (PRJNA400107) that are negative for *tet*(X4). The negative isolates were non-duplicates and originated from human stool samples collected from healthy individuals in 2016 across China, in a previous study characterising *mcr-1* carriage in humans^11^. A core-genome SNP tree was constructed and visualised, as described above.

### Identification of STs, AMR, and virulence genes

Multi-locus sequence types (MLST) were assigned according to the *E. coli* MLST database^25^ by mapping cleaned reads to the alleles using SRST2^26^. A minimum spanning tree based on generated sequence types was constructed in BioNumerics version 7.0 (Applied Maths) and used to differentiate the isolates in terms of their origin. Given the clinical importance of AMR and virulence in *E. coli*, a targeted analysis of all known acquired AMR genes and virulence-factor-associated genes was performed within our genomic dataset. These genes were screened using abricate against the ResFinder 2.1^27^ and vfdb^28^ databases (>90% identity). The classification of each strain into phylogenetic groups was performed according to the scheme described previously^29^.

### *tet*(X4) gene location, environment and plasmid typing

We determined the plasmid or chromosome location of the *tet*(X4) gene in the selected isolates by ONT long-read sequencing analysis. Contigs harbouring *tet*(X4) were searched and extracted from the assemblies using contig-puller (https://github.com/kwongj/contig-puller) and checked for cyclization. By searching against PlasmidFinder^30^ (>95% identity and >90% coverage), circular contigs harbouring plasmid replicons were considered plasmid-borne and circular contigs with no replicons detected were considered circular intermediates. All contigs were submitted to PATRIC^31^ for the putative coding sequences of the genes flanking *tet*(X4).

### Statistics and reproducibility

Descriptive analysis on prevalence, 95% confidence interval and median calculation were performed using functions provided in Excel 2019 (Microsoft). Exact sample sizes for each group were described in Table 1. Source data used to plot Figs. 2–5, Supplementary Figs. 1 and 2 are archived in Supplementary Data 2.

### Reporting Summary

Further information on research design is available in the Nature Research Reporting Summary linked to this article.

## Supplementary information

Supplementary Information

Supplementary Data 1

Supplementary Data 2

Description of Additional Supplementary Files

Reporting Summary

## Data Availability

Data supporting the findings of this study are included in this article and in the Supplementary Information. Genome assemblies of the 95 *tet*(X4)-positive *E. coli* have been deposited in the NCBI and are registered under BioProject accession no. PRJNA625924. All data are available from the corresponding authors upon reasonable request.
