# Supplementary material for: Genomic epidemiology of animal-derived tigecycline-resistant Escherichia coli across China reveals recent endemic plasmid-encoded tet(X4) gene
Source: Commun Biol. 2020 Jul 31;3:412. doi: 10.1038/s42003-020-01148-0 (PMC7395754; doi:10.1038/s42003-020-01148-0)
Supplement: Supplementary file 1 — Supplementary Information [file 42003_2020_1148_MOESM1_ESM.pdf]

Supplementary Figures

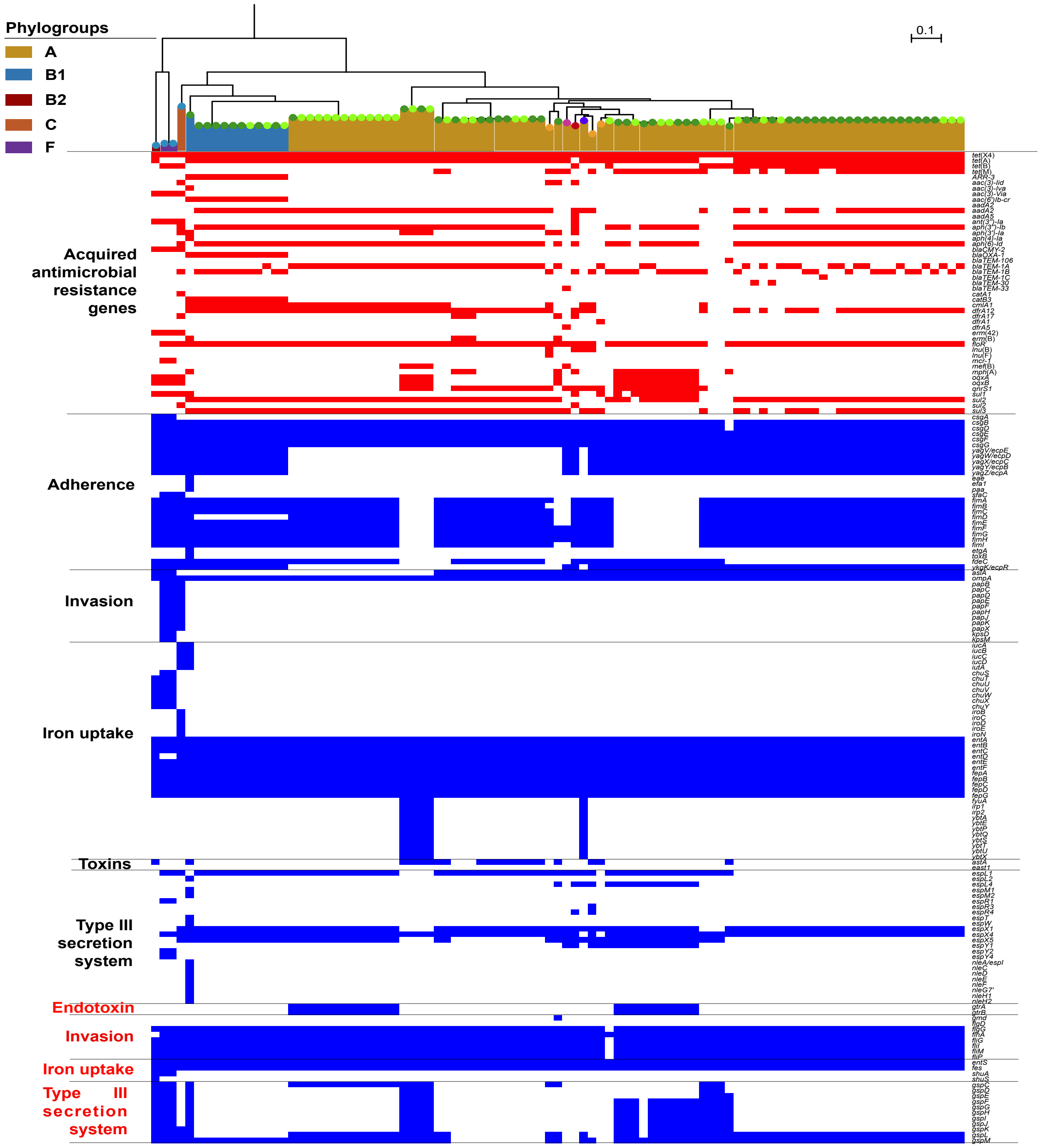

Supplementary Figure S1. Incidence of antibiotic resistance genes and virulence factor- associated genes in the 95 *tet*(X4)-positive *E. coli*. Each isolate is labelled on the node with a coloured dot representing its location (as indicated in figure 2). Squares indicate the presence of antibiotic resistance genes (red) and virulence factor-associated genes (blue) in each isolate. Genes are labelled on the left side and grouped on the right side. The red text represents as-yet-uncharacterised virulence factors in *E. coli* that have ever been found in bacteria species other than *E. coli*.

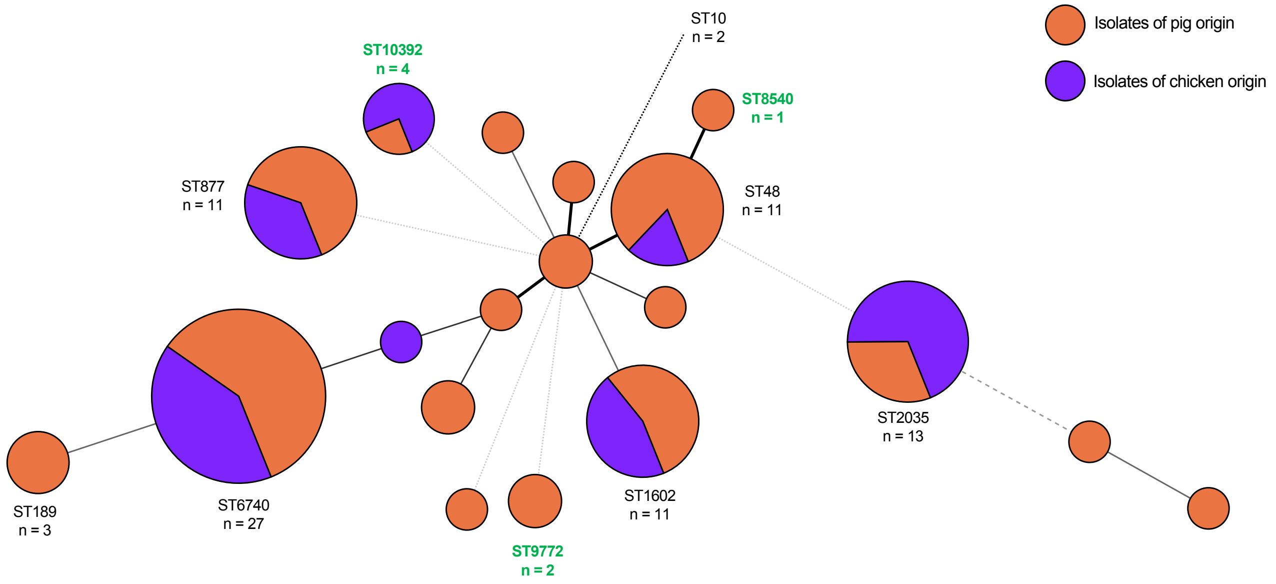

Supplementary Figure S2. Minimum spanning tree of the 95 *tet*(X4)-positive *E. coli* by MLST type and gene allele profile. Each node represents a single ST differing in size proportional to the number of isolates. Selected nodes are labelled with the corresponding ST and number of isolates; nodes labelled in green represent novel STs identified in this study.

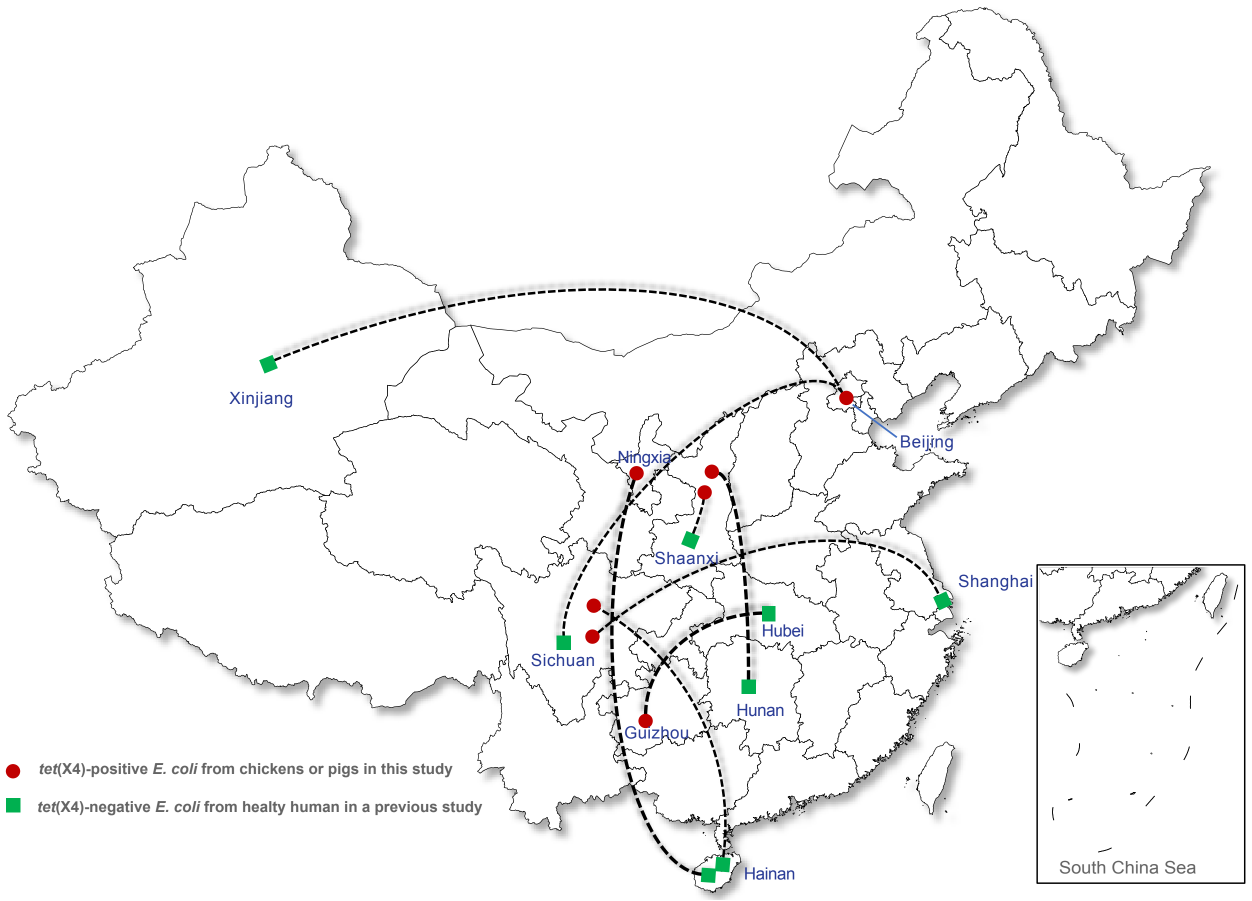

Supplementary Figure S3. Map of China showing the geographical locations of the *E. coli* isolates with high genetic similarities. Red dots represent the *tet*(X4)-positive *E. coli* isolates from food animals in the present study, green squares represent *tet*(X4)-negative *E. coli* isolates from healthy humans in a previous study, and the dotted lines connecting isolates with high genetic similarities (39–389 SNPs).
