# Supplementary material for: Genomic epidemiology of animal-derived tigecycline-resistant Escherichia coli across China reveals recent endemic plasmid-encoded tet(X4) gene
Source: Commun Biol. 2020 Jul 31;3:412. doi: 10.1038/s42003-020-01148-0 (PMC7395754; doi:10.1038/s42003-020-01148-0)
Supplement: Supplementary file 4 — Description of Additional Supplementary Files [file 42003_2020_1148_MOESM4_ESM.pdf]

Description of Additional Supplementary Files

File Name: **Supplementary Data 1**

Description: Minimum inhibitory concentration values of the 95 *tet*(X4)-positive *E. coli*.

File Name: **Supplementary Data 2**

Description: Characterization of the 95 sequenced *tet*(X4)-positive *E. coli*. Source data used to plot Figs. 2, 3, 4, 5, Supplementary Fig. 1 and Supplementary Fig. 2
