# Supplementary material for: Genomic epidemiology of animal-derived tigecycline-resistant Escherichia coli across China reveals recent endemic plasmid-encoded tet(X4) gene
Source: Commun Biol. 2020 Jul 31;3:412. doi: 10.1038/s42003-020-01148-0 (PMC7395754; doi:10.1038/s42003-020-01148-0)
Supplement: Supplementary file 5 — Reporting Summary [file 42003_2020_1148_MOESM5_ESM.pdf]

nature research

Corresponding author(s): Congming Wu, Shixin Xu

Last updated by author(s): Jun 12, 2020

Reporting Summary

Nature Research wishes to improve the reproducibility of the work that we publish. This form provides structure for consistency and transparency in reporting. For further information on Nature Research policies, see Authors & Referees and the Editorial Policy Checklist.

Statistics

For all statistical analyses, confirm that the following items are present in the figure legend, table legend, main text, or Methods section.

n/a	Confirmed	
☐	☒	The exact sample size (n) for each experimental group/condition, given as a discrete number and unit of measurement
☒	☐	A statement on whether measurements were taken from distinct samples or whether the same sample was measured repeatedly
☒	☐	The statistical test(s) used AND whether they are one- or two-sided<br>*Only common tests should be described solely by name; describe more complex techniques in the Methods section.*
☒	☐	A description of all covariates tested
☒	☐	A description of any assumptions or corrections, such as tests of normality and adjustment for multiple comparisons
☐	☒	A full description of the statistical parameters including central tendency (e.g. means) or other basic estimates (e.g. regression coefficient) AND variation (e.g. standard deviation) or associated estimates of uncertainty (e.g. confidence intervals)
☒	☐	For null hypothesis testing, the test statistic (e.g. F, t, r) with confidence intervals, effect sizes, degrees of freedom and P value noted<br>*Give P values as exact values whenever suitable.*
☒	☐	For Bayesian analysis, information on the choice of priors and Markov chain Monte Carlo settings
☒	☐	For hierarchical and complex designs, identification of the appropriate level for tests and full reporting of outcomes
☒	☐	Estimates of effect sizes (e.g. Cohen's d, Pearson's r), indicating how they were calculated

Our web collection on statistics for biologists contains articles on many of the points above.

Software and code

Policy information about availability of computer code

Data collection	All statistic data were collected and organized using Microsoft Excel for Mac 2019.
Data analysis	All statistic data were organized and calculated using Microsoft Excel for Mac 2019. The draft genomes were assembled using SPAdes version 3.9.0, and the hybrid de novo assembly combining Illumina short reads and Oxford Nanopore Technologies long reads was conducted using unicycler version 0.4.8; MLST typing were assigned using SRST2 toolkit version 0.2.0, and the minimum spanning tree based on generated MLST types was constructed in BioNumerics version 7.0; Antibiotic resistance genes and virulence-associated genes were determined using abricate version 0.9.8 against the ResFinder (01 October 2019) and vfdb (01 November 2019) databases; Core-genome SNPs based Neighbour-Joining phylogenetic trees were constructed using Parsnp version 1.2 in the Harvest package version 1.1.2, and visualized by iTOL v5, the generated figures were further edited by Adobe Illustrator 2019; Contigs harboring tet(X4) were searched and extracted from the assemblies using contig-puller (https://github.com/kwongj/contig-puller) and checked for plasmid replicons using abricate version 0.9.8 against the PlasmidFinder database (09 Feburary 2018); All contigs were annotated for the putative coding sequences using PATRIC version 3.6.2; The classification of each strain into phylogenetic groups was performed using ClermonTyping (https://github.com/A-BN/ClermonTyping).

For manuscripts utilizing custom algorithms or software that are central to the research but not yet described in published literature, software must be made available to editors/reviewers. We strongly encourage code deposition in a community repository (e.g. GitHub). See the Nature Research guidelines for submitting code & software for further information.

Data

Policy information about availability of data

All manuscripts must include a data availability statement. This statement should provide the following information, where applicable:

- Accession codes, unique identifiers, or web links for publicly available datasets
- A list of figures that have associated raw data
- A description of any restrictions on data availability

Data supporting the findings of this study are included in this article and in the Supplementary Information. Genome assemblies of the 95 tet(X4)-positive E. coli have been deposited in the NCBI and are registered under BioProject accession no. PRJNA625924. All data are available from the corresponding authors upon reasonable request.

Field-specific reporting

Please select the one below that is the best fit for your research. If you are not sure, read the appropriate sections before making your selection.

☒ Life sciences ☐ Behavioural & social sciences ☐ Ecological, evolutionary & environmental sciences

For a reference copy of the document with all sections, see nature.com/documents/nr-reporting-summary-flat.pdf

Life sciences study design

All studies must disclose on these points even when the disclosure is negative.

Sample size	This article describes a cross-sectional study to investigate the prevalence of tet(X4)-carrying E. coli from pigs and chickens in China in 2018. The study includes a large E. coli collection available from food animals across 22 (out of 34) provinces and municipalities in China, which is based on isolates from China's national AMR surveillance program in zoonotic bacteria.
Data exclusions	The surveillance system has received a total of 3124 E. coli isolates from 233 farms and slaughterhouses across 22 of the 34 provinces and municipalities in China. Because there was a large variation in the number of isolates from each farm and slaughterhouse, we randomly selected about 15 E. coli isolates from each farm or slaughterhouse in order to minimise the sample bias in the surveillance data, and this resulted in a total of 2,475 E. coli isolated from 166 farms and slaughterhouses distributed across the country (Figure 1 and Table 1).
Replication	This study is a point-prevalence cross-sectional study examining the prevalence of tet(X4)-carrying E. coli from food animals at a specific point of time in a specific location (national scale of China), the mean positive percentage tet(X4) rate was 4.9% (95% CI: 3.7%-6.2%) and 2.8% (95% CI: 2.0%-3.9%) from pig and chicken isolates, respectively, the high level of 95% CI indicates that the reproducibility of this finding can be reliably produced. For each PCR test, appropriate controls were run with each batch. Positivity has also been further confirmed by sequencing and this correlation was 100%. Exact sample sizes for each group were described in Table 1. Source data used to plot Figs. 2, 3, 4, 5, Supplementary Fig. 1 and Supplementary Fig. 2 are archived in Supplementary Table 2.
Randomization	The participation of farms and slaughterhouses from each province or municipality in the national surveillance system is on a voluntary basis. From each farm or slaughterhouse, around 15 E. coli isolates randomly selected into the current study. The E. coli isolates were identified without any targeted AMR phenotypes.
Blinding	The chicken cloacal swabs and pig fecal swabs were collected and identified by researchers in provincial laboratory or institute certified in joining the surveillance program. The isolates were initially collected for routine AMR surveillance purposes and not specifically for this study. The authors had no direct contact with the researchers in provincial laboratory or institute, lab experimenters and data analysis not could the authors influence sample enrolment.

Reporting for specific materials, systems and methods

We require information from authors about some types of materials, experimental systems and methods used in many studies. Here, indicate whether each material, system or method listed is relevant to your study. If you are not sure if a list item applies to your research, read the appropriate section before selecting a response.

Materials & experimental systems

n/a	Involved in the study	
☒	☐	Antibodies
☒	☐	Eukaryotic cell lines
☒	☐	Palaeontology
☐	☒	Animals and other organisms
☒	☐	Human research participants
☒	☐	Clinical data

Methods

n/a	Involved in the study	
☒	☐	ChIP-seq
☒	☐	Flow cytometry
☒	☐	MRI-based neuroimaging

Animals and other organisms

Policy information about studies involving animals; ARRIVE guidelines recommended for reporting animal research

Laboratory animals	The study did not involve laboratory animals.
Wild animals	The study did not involve wild animals.
Field-collected samples	The study is based on E. coli collection from China's national AMR surveillance program in zoonotic bacteria. The E. coli isolates were collected and submitted by provincial laboratories or institutes certified as part of the surveillance programme. The current study is conducted based on the E. coli dataset, other than field collected samples.
Ethics oversight	Since no live vertebrates were used in this study, the included E. coli collection is exempt from the IACUC approval process.

Note that full information on the approval of the study protocol must also be provided in the manuscript.
